# Characterization of Environmental Health Inequalities Due to Polyaromatic Hydrocarbon Exposure in France

**DOI:** 10.3390/ijerph15122680

**Published:** 2018-11-28

**Authors:** Despoina Ioannidou, Laure Malherbe, Maxime Beauchamp, Nicolas P.A. Saby, Roseline Bonnard, Julien Caudeville

**Affiliations:** 1 National Institute for industrial Environment and Risks (INERIS), Verneuil-en-Halatte 60550, France; idespoin@windowslive.com (D.I.); laure.malherbe@ineris.fr (L.M.); maxime.beauchamp76@gmail.com (M.B.); roseline.bonnard@ineris.fr (R.B.); 2 National Conservatory of Arts and Crafts (CNAM), Paris 75003, France; 3 National Institute of Agronomic Research (INRA), US 1106, Orleans 45000, France; nicolas.saby@inra.fr

**Keywords:** environmental health inequalities, spatial exposure, polycyclic aromatic hydrocarbon

## Abstract

Reducing environmental health inequalities has become a major focus of public health efforts in France, as evidenced by the French action plans for health and the environment. To evaluate environmental inequalities, routine monitoring networks provide a valuable source of data on environmental contamination, which can be used in integrated assessments, to identify overexposed populations and prioritize actions. However, available databases generally do not meet sufficient spatial representativeness to characterize population exposure, as they are usually not assembled for this specific purpose. The aim of this study was to develop geoprocessing procedures and statistical methods to build spatial environmental variables (water, air, soil, and food pollutant concentrations) at a fine resolution, and provide appropriate input for the exposure modelling. Those methods were designed to combine in situ monitoring data with correlated auxiliary information (for example, atmospheric emissions, population, and altitude), in order to better represent the variability of the environmental compartment quality. The MODUL’ERS multimedia exposure model developed by INERIS (French Institute for industrial Environment and Risks) was then used to assess the transfer of substances from the environment to humans, through inhalation and ingestion pathway characterization. We applied the methodology to a carcinogenic Polycyclic Aromatic Hydrocarbon substance, benzo[a]pyrene(B[a]P), to map spatialized exposure indicators, at the national scale. The largest environmental contribution corresponded to the ingestion pathway. Data processing algorithms and calculation of exposure will be integrated into the French coordinated integrated environment and health platform PLAINE (PLteforme intégrée d’Analyse des INégalités Environnementales) which has been developed to map and analyze environmental health inequalities.

## 1. Introduction

Environmental health inequalities refer to the general differences in environmental health conditions. It refers to the recognition of disparities between territories or populations concerning environmental exposure. In some areas, people are more likely to be exposed to the negative effects of air, soil, or water pollution. This issue has been identified as a priority for French policies, as evidenced by the emergence of the French plans for health and environment. Specifically, the third plan (2015–2019) highlights the need to establish a methodology to identify and reduce environmental health inequalities. Contamination process is extremely complex and variable through space and time, with localized multiple sources. Exposure assessment is defined as the science that describes how an individual or a population can come in contact with contaminants, and it provides an adapted framework to characterize environmental inequalities. The analysis of spatial environmental data to assess exposure, is a fundamental element that first requires different scientific limitations to be overcome, such as the linkage of the several databases to describe the global source-effect chain [1].

Some studies integrate georeferenced measure monitoring or modelling data to estimate the exposure dose, and may include studies on various single environmental media, such as soil [2], water [3], and air [4]. In the context of mapping the environmental inequalities, additional requirements are needed in the exposure assessment processes:

- Characterize the principal exposure pathways.
- Build realistic scenarios that integrate the past and present sources.
- Describe the phenomena at a fine temporal and spatial resolution.

Human exposure to different substances occurs from the transfers in the various environmental compartments. For example, Polycyclic Aromatic Hydrocarbons (PAHs) enter the human body through ingestion of food, either due to their presence in uncooked food or due to the cooking process [5,6]. Important pathways include inhalation of ambient air and drinking water. Evidence suggests that exposure to PAH mixtures can induce carcinogenicity [7,8]. It is, therefore, crucial to identify and monitor potentially exposed populations, especially for benzo[a]pyrene (B[a]P), as it has been characterized as a Group 1 carcinogen for humans (International Agency for Research on Cancer).

Multimedia exposure models can be used to consider the contribution of each exposure pathway in the estimation, to finely characterize the total exposure [9]. The use of such a model allows to establish a quantitative framework for evaluating the substance transfer in the environment and the exposure compartments, as well as analyzing the population exposure. Consequently, the assessment results can provide a robust and reliable basis for the management of environmental health inequalities. The different multimedia model spatial scales have been discussed in detail by McKone and MacLeod (2003) [10]. As pointed out by the authors, multimedia models have evolved from evaluative models to regional mass-balance models, multi-region models, and global models. The very fine resolution does not correspond to enough detailed knowledge and does not reflect local exposure variations, with sufficient accuracy. Moreover, these fate and transport models have failed to capture the background exposure and past sources, since historical emission sources are not available for a sufficiently long period. This issue could be partially overcome by using monitoring networks that provide good quality data for the characterization of exposure pathways. The multimedia exposure model MODUL’ERS was initially developed by INERIS (French Institute for industrial Environment and Risks) [11,12] to assess the transfer of contaminants from the environment (air, soil, and water), through the local food chain, to individual exposure. This study has added a spatial dimension to the model using Geographic Information System (GIS) and has been adapted to the French regional and national monitoring available databases.

At a regional scale and fine resolution, the direct use of environmental quality measurements is not appropriate for assessing the exposure levels of a contaminant, over a continuous area, as it can lead to inappropriate exposure characterization. To reduce this problem, several more sophisticated methods of spatial analysis have been developed, to improve the data resolution and characterize the associated uncertainty [13,14]. Geostatistical approaches are often employed to spatialize environmental monitoring data. To obtain spatially exhaustive data, when sampling density is limited, information from the auxiliary variables can be included to avoid exposure media contribution distortion, in the total exposure [15,16,17]. Another ubiquitous issue in using environmental databases is the presence of values, under the limit of detection. The need to address this issue has been previously highlighted [3,18]. Usual approaches to treat observations under the detection limit (DL) or quantification limit (QL), include substitution of the missing values with a constant such as: 0, DL/2, ... [4,19]. This method proved to be an effective cost-wise option when the percentage of observations under the detection limit is less than 5% [20]. Otherwise, they introduce greater bias, and methods like multiple imputations are shown to be more adequate [20]. Spatial pattern is also an important aspect when calculating the exposure dose. Oversimplifications, such as assigning an average value to a large region, can lead to misestimation [21,22], and consequently, a misclassification of the exposure [2].

The present study aims to harmonize the data coming from different sources and explore spatial statistical methods to improve the limited resolution of measurements, in the three environmental compartments (air, water, and soil), and to set the basis for a robust multimedia exposure assessment due to Benzo[a]pyrene (B[a]P), in France. The above issues are addressed by employing statistical and geostatistical methods to spatialize concentration measurements of B[a]P in France in these three environmental compartments. A multiple imputation method that takes advantage of the temporal aspect and the linear relationships between B[a]P and other PAH substances, is employed to take the values under the detection limit into account. To address the issue of limited number of observations and representativeness, appropriate spatial auxiliary variables are constructed. Finally, estimated concentrations of B[a]P, in the different compartments, are integrated into a multimedia exposure model to yield the aggregated exposure estimation and associated environmental determinant.

## 2. Materials and Methods 

### 2.1. Data Description and Exposure Model

No statewide data were available to provide direct information on exposure. Exposure generally involves transfer of chemicals from a source through the environment (air, water, soil, and food) to an individual or population. Environmental concentrations of B[a]P were obtained from available monitoring databases. Overlaying data from several sources were selected, according to the availability, spatial coverage, and their relevancy in the framework of exposure evaluation and environmental health inequalities (Table 1).

**Figure 1:**
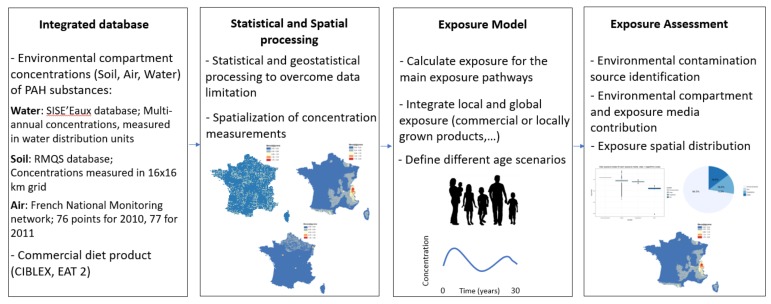
Illustration of the exposure assessment process.

For the purposes of the study, data related to pollutant sources, releases, and environmental concentrations were used to build indicators of potential human exposures to pollutants. Statistical and geostatistical methods were developed to spatialize annual or annual average concentrations of B[a]P in France, in the three environmental compartments (air, water, and soil) on a referent grid (9 km^2^ grid). A multimedia model developed by INERIS (MODUL’ERS) and operating in a GIS environment was employed for quantifying the human exposure to toxic substances and analyze the environmental determinants. An illustration of the different steps of this process can be found in Figure 1. 

#### 2.1.1. Water Compartment

The presence of PAH substances in drinking water is observed either due to the raw water composition or due to the coating of the drinking water distribution pipes. As drinking water is an important factor of human exposure to potentially toxic substances, characterizing its quality is an essential step when estimating the total exposure to various substances. The concentration measurements of PAH substances in drinking water in France were analyzed from the French regulatory monitoring database, SISE’Eaux (Water Environmental Health Information System) [24]. Data were collected in the framework of monitoring and evaluating the quality of the distributed water by the French Ministry of Health. The concentrations were measured in the 24,655 distribution units (UDI) that serve all 36,000 municipalities, constituting the water distribution network in France. The latter was constructed in such way that a single distribution unit could serve more than one municipality and one municipality could be served by more than one unit. In total, 8 PAH substances were included in the database—anthracene, benzo[a]pyrene, benzo[b]fluoranthen, benzo[k]fluoranthen, fluoranthen, indeno[1,2,3-cd]pyren, and napthalen. The measurements were collected during the years 2000 and 2012, with no periodicity. Moreover, the total number of observations for each substance varied, since not all substances were measured with the same frequency in the different distribution units. As often observed in environmental measurements, the rate of observations, under the detection limit, was quite high. Specifically, the measurements under the detection limit for B[a]P accounted for 88.1% of the total number of observations (less than 2 million). Therefore, before spatializing the data, a crucial step of the process was to account for these observations to extract the maximum information possible.

#### 2.1.2. Soil Compartment

Deposition of PAHs near emission sources, such as traffic, production of creosote, cement, asphalt, and the petrochemical industry, can display complex spatial patterns, depending on the prevailing weather conditions (e.g., wind direction, and wet and dry deposition), the local topography, and the characteristics of the source. Soil can become a reservoir for non-degraded PAHs, and interactions with some soil constituents can favor accumulation. Therefore, soil is a key environmental compartment regulating the fate and recording the evidence of the contamination process [23].

The objective here was to estimate the concentration of B[a]P in the topsoil on the 9 km^2^ reference grid, considering data from different available sources to increase the data resolution. Available soil concentration measurements were derived from the Soil Quality Monitoring Network [23]. The database contains concentrations of 16 PAH substances in the topsoil (0–30 cm), for 1710 sample locations in a systematic 16 × 16 km regular grid, across the 550,000 km^2^ of the French metropolitan territory. The number of measurements under the detection limit accounted, on average, for 67, 8% of the total observations.

Localization of the polluted sites, in France, are available from the BASOL database (Potentially Contaminated Soil Database, French Ministry in charge of the environment) and are used for the construction of an auxiliary variable. This database constitutes an inventory of the contaminated sites, to orientate preventive or curative actions of the administrative authorities, and it contains information on substances present in the contaminated sites. To map the PAH substances content in soil, at a final resolution, we implemented a digital soil mapping approach [17]. For this, several potentially interesting environmental covariates were considered. The environmental covariates included fourteen exhaustive covariates in relation to the soil forming factors, available in 500 m resolution [25].

#### 2.1.3. Air Compartment

Ambient air contamination occurs mainly due to incomplete combustion processes, in the environment, of various sources (industry, road traffic, wood burning, etc.). PAHs are more persistent in urban and highly industrialized areas, but their presence in the air can also be due to atmospheric long-range transport [10,26]. 

PAH concentration data in the air compartment were available from the French air quality database, in which data collected in each French region, in the context of regulatory surveillance, were collected. They consist of annual mean concentrations aggregated from daily or weekly data, for the years 2010–2011. The seventy-six monitoring stations were categorized according to their location (defined as rural, suburban, or urban) and the type of influence they were submitted to (background, traffic, industrial). Cumulative annual emissions of the PAH substances for 2007 (National Spatialized Inventory), including emissions from different sources (industrial combustion, use of solvents, urban, traffic, etc.) were available in a 0.01° × 0.01° grid, also for the French territory.

#### 2.1.4. Spatial Exposure Model

To estimate the aggregated exposure to PAH in France, national and local data needed to be integrated in a common calculation framework. A modeling platform with a GIS interface was set in place to calculate the exposures due to ingestion and inhalation. It integrated the available data on a reference grid, after the individual processing of each element of the source-effect chain. The relevant data included:

- spatialized environmental concentrations from the available environmental data for each compartment (air, water, soil);
- population data, describing population density and dietary habits of the homogeneous population subgroups of reference;
- exposure media concentrations and exposure doses from multimedia modeling.

The MODUL’ERS multimedia exposure model [11,12] was used to assess the transfer of contaminants, from the environment, to individual exposure, through the local food chain. It combines assumptions and concentration measurements of environmental agents in air, water, soil, and food, with data on inhalation and ingestion rates, to produce an estimate of intake of the environmental agent. To describe the ingestion pathway, it was necessary to define the body weight, the food behavior, the water consumption, the soil quantity ingested, and the auto-consumption factor of the considered population. In the context of environmental health inequalities cartography, the exposure scenarios needed to allow the comparison among the different defined groups. To describe more realistic exposure scenarios, subgroups in terms of age were defined (children 0–17 of age and adults of age 17–70). These two age-class exposure variables could easily be determined from the French national studies, and corresponded to a compromise between the calculation time and age class variability relevance. Receptor parameters were built to account for inter- and infra-regional disparities in the population ingestion comportment. The consumption rates of the different food categories were derived from the French database CIBLEX (French exposure variables database), which is a compilation of different available exposure factors, at a regional scale.

To assess the exposure dose, predicted concentrations of the substance of interest, from local exposure media and national data for commercial foodstuffs, were integrated in the model. The pathways of exposure considered here were:

- inhalation of local air;
- ingestion of locally produced vegetables;
- ingestion of commercial product;
- ingestion of soil;
- ingestion of drinking water.

The estimation of concentrations in the homegrown products was done by combining air, soil, and transfer models. The exposure associated with the consumption of products, outside the study zone, were also included. The model’s equations were adapted, considering the local and chronic nature of exposure. The spatially explicit model was described in Caudeville et al., 2012 [1]. Equations have been adapted for the carcinogenic effect. The model was used to calculate the Lifetime Average Daily Doses (LADDs) and an Excess of Individual Risk (EIR). The results were then georeferenced in a GIS framework, to yield the exposure maps.

### 2.2. Spatial Data Processing 

To assess the exposure with adequate precision, the construction of a high quality spatial database was a prerequisite. In this section we present the methodology developed to spatialize the B[a]P concentration measurements in the three environmental compartments, to be used as input to the exposure model. 

#### 2.2.1. Water Compartment 

##### Spatialization of Observations, Including Values under the Detection Limit 

Spatialization of observations, including values under the detection limit was carried out by spatializing the multi-annual average concentrations, at the municipality level (European NUTS – Nomenclature of Territorial Units for Statistics- 4). There were three issues that needed to be addressed:

- Including the observations under the detection limit (left censored data), without introducing great bias in the process.
- Accounting for the temporally irregular sampling.
- Estimating the mean multi-annual concentration by municipality, considering the network complexity.

Left censored data have been a ubiquitous problem in environmental sciences. Different approaches have been proposed, the most simplistic one being the substitution of under the detection limit measurement with a constant. However, this kind of method has been proven to introduce great bias in the estimation, as well as under-estimating the variance when the percentage of left-censored data was well over 5% [4].

An alternative was the use of multiple imputation. The idea was to replace each missing value (non-detected) with a set of plausible values that represent the uncertainty about the right value to impute, instead of filling in a single value for each missing value. These multiple imputed datasets were then analyzed using the standard procedures for complete data and combining the results from these analyses. Usually in environmental science, a model-based imputation is suggested [27]. Model-based imputation consists of generating random observations below the detection limit, from a distribution estimated from the detected sample values, and analyzing them as complete cases. Such a method would not work with these data, since there were numerous facilities for which most or all the measurements were beneath the detection limit, making it unreliable to fit a distribution for each facility. 

Honaker [28], has proposed a bootstrap-based expectation-maximization (EM) algorithm to perform the multiple imputation. This method consists of creating multiple “complete” datasets, that could then be analyzed as complete cases. The algorithm treats time series-cross sectional data, by using the Expectation-Minimization (EM) algorithm on bootstrap samples of the original data, to draw values from the complete data parameters. In this manner, the correlation between these substances could serve for the simulation of the concentration measurements under the detection limit.

Once the imputed datasets were retained, the estimation of the concentrations for each water distribution unit was carried out as the multi-annual weighted mean. The weights were calculated by the segments of the influence method [16]. According to this method, a median was traced between two neighbor events, then a weight was assigned to each realization. This weight was equal to the number of days between the two included segments, divided by the total number of days between the starting date (1 January 2000), and the finish date (31 December 2012). Finally, the results were spatialized by georeferencing the concentrations, for each municipality, to the 3 × 3 km grid of reference. As it is not possible to directly test its performance, we applied the imputation method on a more complete pollutant dataset (water arsenic concentration correlated with selenium pollutant). The new dataset for the simulation was chosen to correspond to different pollutants of the same water information system. We artificially substituted the known concentrations with different DL thresholds (3 µg·L^−1^ and 5 µg·L^−1^ resulting, respectively, in 82% and 88% of the measurements under the detection limit). We examined the ‘bias’ using Absolute Mean Error (AME) and Root-Squared Mean Error (RMSE) statistics to estimate how close the imputed values were to the actual observed values. To demonstrate the properties of the method, we compared statistics from those obtained for the naive methods for handling data of this nature, including a simple substitution with DL/√2 and DL/2.

#### 2.2.2. Soil Compartment

##### Refining the Estimation with Auxiliary Variables 

Regression followed by kriging of the residual [29] was employed to interpolate the concentration measurements between the sample locations. This method was popular when predicting spatial concentrations of pollutants in soil and contamination mapping [30,31,32], as it allowed to integrate secondary information from auxiliary variables in the prediction, exploiting linear relationships between the variables. The candidate covariates for the model included an indicator variable representing the probability of exceeding the detection threshold, a function of the distance between the measurements and the polluted sites, and several soil properties that might affect the spatial distribution of PAH in soil. Knowledge of the processes controlling the spatial variation of the property could be included in the model by selecting appropriate covariates. For example, we expected the variation of B[a]P in French soils to be influenced by the geological setting, inputs from anthropogenic activities, such as agriculture, industry and mining, and the transport and deposition of these elements from these sources. Therefore, covariates reflecting those processes, namely a classification of parent material, a classification of land use, the average annual precipitation, and the average annual potential evapotranspiration, were introduced in the kriging. 

In order to deal with values under the detection limit (DL) and account for local uncertainty, the probability of the measurements being under the detection limit was estimated, using the method of Indicator Kriging (IK) [33]. Indicator kriging relies on the discrete transformation of the range of an environmental attribute by a threshold (in this case, the detection limit), and transforms each observation into a vector of indicators of non-exceedance of the threshold. Kriging is then used to interpolate the transformed values. The interpolated values were calculated as the probability of exceeding the detection limit. This probability variable was then used as a predictor in residual kriging. The second predictor that could be considered for the residual kriging, was the distance between the locations and the pre-identified polluted sites. The variable was constructed using the BASOL database. First, a buffer was built around the polluted sites. Then the distance between the polluted site and the monitoring locations that fell inside the buffer, was calculated. Different buffer radii was tested to find the optimal inverse distance decay function, using the Akaike criterion [34]. 

Once the auxiliary variables were constructed, residual kriging was then employed to interpolate the concentration measurements. Typically, the residuals of a previously fitted linear regression were interpolated using ordinary kriging. This way it was possible to include information from external explanatory variables to the kriging process. An alternative to the linear regression was the use of a machine learning algorithm to account for complex and nonlinear relationships between the variable of interest and environmental factors. In this study, the application of the method of random forests has been investigated. The random forest regression approach consists in producing multiple regression trees and then combining them to give a single consensus prediction [35]. Random forests were more adequate in describing complex, nonlinear relationships, and have been proven to outperform the linear regression [36]. Moreover, they do not require any assumptions about the relationship between the variable of interest and the predictors. 

The candidate covariates to be added in the model included the two auxiliary variables constructed, in addition to the fourteen environmental covariates which describe the soil and the geological context. The importance of the variables was assessed in the random forest framework, while the covariates selected were the ones that minimize the out-of-the-bag (OOB) error rate. Once the random forest regression was fitted, the variogram was modeled, using a spherical model with three standard parameters, nugget, partial sill, and range. The final performance was evaluated with cross-validation. 

#### 2.2.3. Air Compartment

##### Defining a Correlated Auxiliary Variable to Compensate for a Limited Number of Observations 

The main problem with the available measurements was the limited number of observations. Thus, interpolating the concentrations over France, by ordinary kriging, could lead to a misrepresentation of the phenomenon. However, it was possible to refine the estimation by including information from an auxiliary variable. Different kriging approaches, in a multivariate context, have been implemented in France and Europe, to map the atmospheric concentrations—linear regression followed by residual kriging as implemented for the soil compartment, cokriging [37,38]. Regression kriging which has been used and evaluated for many years in the national air quality monitoring and forecasting PREV’AIR system (www.prevair.org) and has been implemented in projects linking air pollution and health effects [39,40], was retained in this study. 

For an accurate spatial prediction, the predictors need to be well-defined. To identify the appropriate predictors, a preliminary study of correlation was made with the measured concentrations. The optimal model was selected as the one that minimized the Mean Error (ME) and the Root Mean Square Error (RMSE), for each station type and for their average. 

To build the predictors, an emission proxy was estimated by fitting a model on data, using aggregated emission grid weighted by distance. A neighborhood matrix was defined to associate grid emissions and distances and measurement observations. The emissions were disaggregated in a 20 km buffer zone, around the PAH measurement locations. The buffer radius was carefully selected considering the decaying correlation between the emissions and the actual measurements, as the distance grew. The auxiliary variable was then defined as a function of the emissions (m), the inverse distance decay (1/d), the altitude (a), and the population (p). Once the model was defined, the variogram was fitted. To model the variogram, an exponential model was employed. Leave-one-out cross validation was then used to assess the final kriging results. 

The results of the two years were finally combined in a single spatialized map, to serve as input for the exposure model. In this way, the estimated concentrations were more representative of the middle-term exposition and less influenced by specificities that could occur in one year but not in the next. For this, a weighted average was employed. The weights were derived for each year, based on the accuracy of the individual predictions, and they were estimated in the following way [41]. 

## 3. Results

### 3.1. Spatialization Results 

#### 3.1.1. Water 

**Figure 2:**
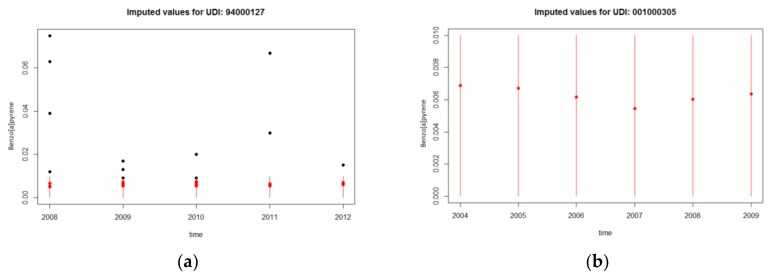
Example of imputations for two water distribution units measured in mg/L; the black points correspond to observed values (>detection limit (DL)), while the red points correspond to the imputation for the same unobserved point, across the datasets obtained from the multiple imputation: (**a**) In this case the imputed values vary between the datasets, due to the existence of multiple observed values before the imputation; (**b**) no observed values were available for this distribution unit, therefore, the imputation was carried out considering only the correlation between the benzo[a]pyrene (B[a]P) and the other Polycyclic Aromatic Hydrocarbons (PAH), therefore, the imputed values between the datasets returned the same values.

B[a]P concentrations under the detection limit were estimated through the multiple imputation method. The strong correlations between substances (85–90%), provided a solid foundation to apply an imputation method for the cross-sectional time series (A Program for Missing Data II: AMELIA II R Package) [28]. For each water distribution unit, the high correlations between the concentrations of the substances and the time trends were also considered for the estimation of the missing values. A minimum of five imputed sets was usually sufficient, however, due to the high percentage of values under the detection limit, eight imputed sets were obtained instead. An example of the concentrations for B[a]P, before and after the imputations for the two units, can be seen in Figure 2. 

**Figure 3:**
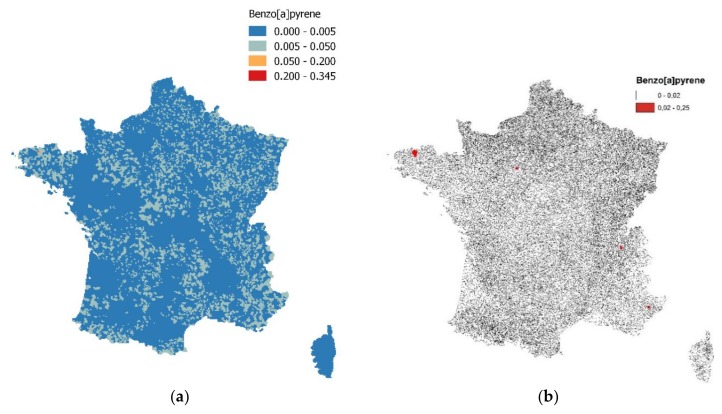
B[a]P concentration maps (mg/L) in water in France, (**a**) for each municipality, and (**b**) with the highlighted highest values.

The variation observed among the imputed datasets depended on the observed data before the imputation. For treatment facilities that had all their monitored values under the detection limit, only correlation between the substances was considered for the imputation. Once the imputed datasets were produced, the multi-year weighted concentration for each treatment facility was calculated and so was the concentration for each municipality, weighted by the population served by each facility in each municipality. Finally, the concentrations were georeferenced on the 3 × 3 km grid to be included in the spatial model. The spatialized results for the municipalities are shown in Figure 3.

Table 2 shows the estimated parameters to compare the difference between the true and the estimated values obtained from our imputation and the naive methods, for the arsenic correlated with selenium pollutant. For the imputation method, the variation that was observed could be partially explained due to the auxiliary variable selected and their weak correlation. However, comparing with the methods of replacing the values under the detection limit, either by DL = 2 or DL = /√2, the imputation method had the smallest absolute mean error. These simulations demonstrate that the imputation method exhibited a better performance in terms of reduced bias.

#### 3.1.2. Soil 

**Figure 4:**
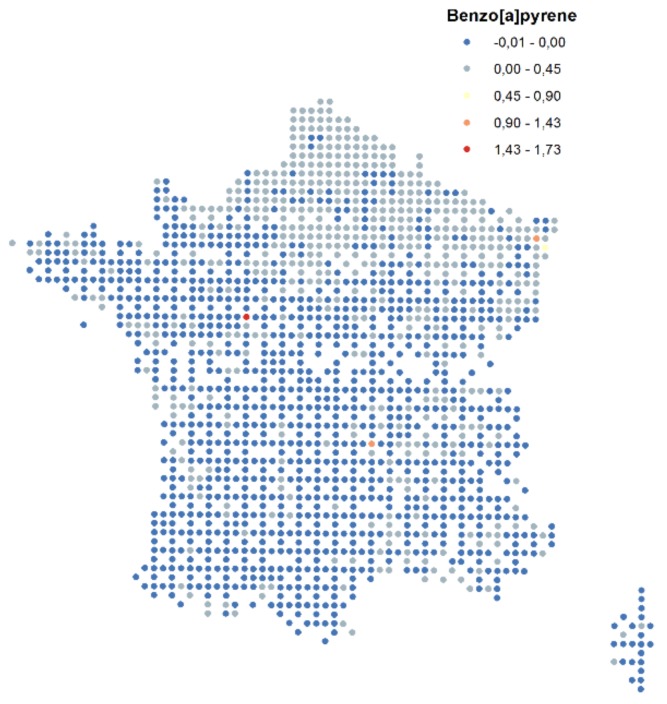
Initial soil data for B[a]P (French soil monitoring network: POP-RMQS); the negative values indicate the values under the detection limit (mg/kg).

The initial topsoil concentration data are presented in Figure 4. 

**Figure 5:**
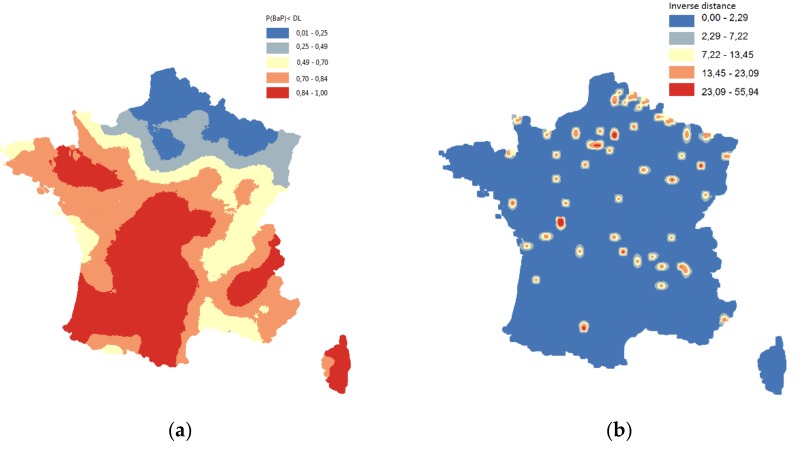
Auxiliary variables to be used in the modeling of B[a]P soil concentration in France. (**a**) Indicator kriging map for the concentrations under the detection limit for B[a]P. (**b**) Source proxy, constructed as a function of inverse distance from the polluted sites (km^−1^).

The probability of a measurement to be above the detection limit (to be used as a covariate in the model) was calculated by the indicator kriging (Figure 5a). The second covariate was the distance from the polluted sites (Figure 5b). 

According to the Akaike criterion, the best buffer to consider is 3 km. Along with those two variables, fourteen soil properties were tested to be included in the random forest model. 

**Figure 6:**
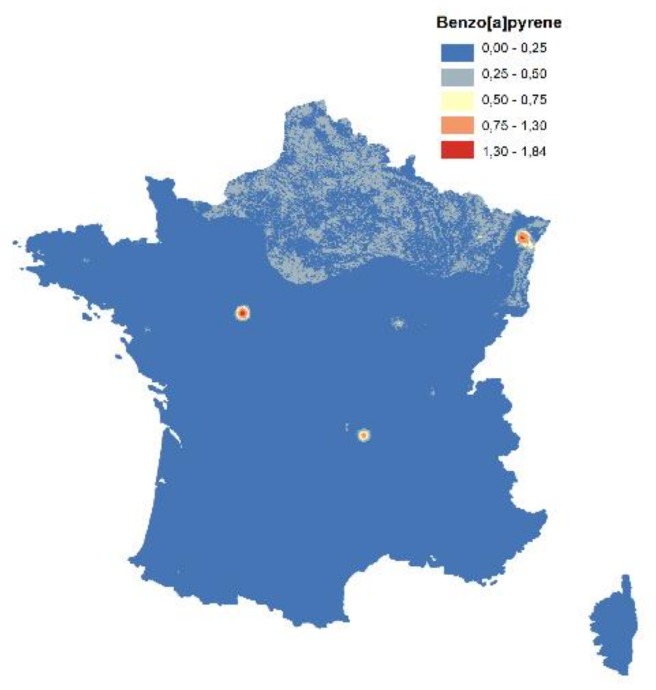
Spatialization of the B[a]P concentrations in soil (mg/kg).

The optimal model was selected to be the one that minimized the Out-Of-the-Bag error. It included eight covariates—the indicator variable describing the probability of exceeding the threshold value, the distance from the polluted sites, and six soil properties. These included, coverage of forest area and semi-natural environment, surface roughness, slope, slope aspect, mean evapotranspiration, and ecoclimate. The final spatialized prediction result for the B[a]P concentrations in soil, are presented in Figure 6. 

Slightly higher values were observed in the north of France, while the highest values were on the hotspots initially measured. The impact of the indicator kriging as an auxiliary variable was particularly evident in this map, however the main trend was heavily influenced by the pattern of the initial data. 

#### 3.1.3. Air

**Figure 7:**
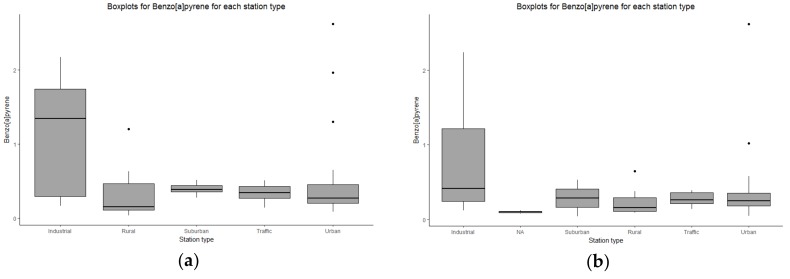
Measured concentrations of the B[a]P (ng/m^3^), by station type, for (**a**) 2010 and (**b**) 2011.

The initial monitoring sites of the PAH atmospheric concentrations were available in seventy-six locations for the year 2010, and seventy-seven locations for the year 2011. Higher concentrations have been mainly observed near industrial emission sources (Figure 7).

**Figure 8:**
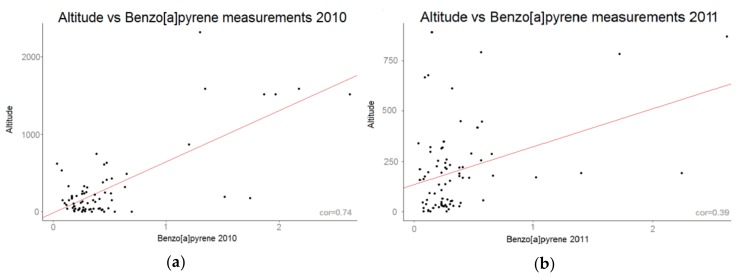
Correlation between altitude (m) and the B[a]P concentration measurements (ng/m^3^) for (**a**) 2010 and (**b**) 2011.

The best auxiliary variable was found to be the one that included the atmospheric emissions, as well as the population and the altitude for the year 2010 and the atmospheric emissions for the year 2011. The correlation between altitude and concentrations, observed for the year 2010 (Figure 8a) was strong, however that was not the case for the concentrations measured in 2011 (Figure 8b), which was why the altitude was only included in the auxiliary variable, for the first map.

The fact that the altitude plays a significant part in the model, seemed to reflect the higher emissions of PAH concentrations due to wood combustion in mountainous regions. Indeed, winter 2010 was colder than winter 2011, which could explain the higher wood combustion rate. 

**Figure 9:**
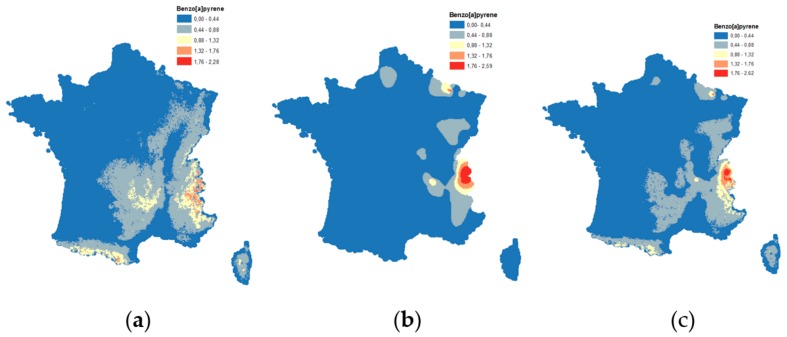
Estimated concentrations of the B[a]P measurements (ng/m^3^) in ambient air, for (**a**) 2010, (**b**) 2011, and (**c**) combined estimated concentration of the B[a]P measurements in ambient air, for both years.

The estimated B[a]P concentrations over the French territory for the two years are presented in Figure 9a,b. As expected, the concentration was higher in the mountain areas, (wood combustion, limited air dispersion). The map for 2011 was smoother than the one of 2010. This was inherited from the auxiliary variable which was different between the two years and included the altitude for 2010. The final results of the two years were then combined in a single map, by a weighted average (Figure 9c).

### 3.2. Spatial Risk Assessment

**Figure 10:**
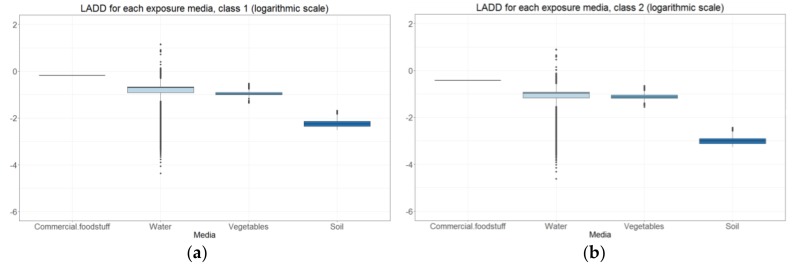
Lifetime Average Daily Doses (LADD) by exposure media for France, measured in mg·kg^−1^·j^−1^: (**a**) Age class 1 (0–17 years); (**b**) age class 2 (17–70 years).

The spatial concentration estimates obtained on the reference grid for the different environmental compartments serve as an input for the multimedia exposure model. The model results allow the analysis of the determinants of exposure, through the estimation of the contributions of the different exposure routes, environmental compartments, and transfers. They could also be used to estimate for which pollutants the risk indicators are high and to identify the populations in potential overexposed areas. Finally, they allow the mapping of the environmental health inequalities, due to exposure through the spatialization of risk indicators. Analyzing the lifetime average daily doses (LADDs), allows the assessment of the variability of the contributions for each ingestion pathway. Figure 10 presents the LADDs for each exposure media for the two age classes (0–17 and 17–70 years old, described in the model) in the totality of the French area for benzo[a]pyrene.

**Figure 11:**
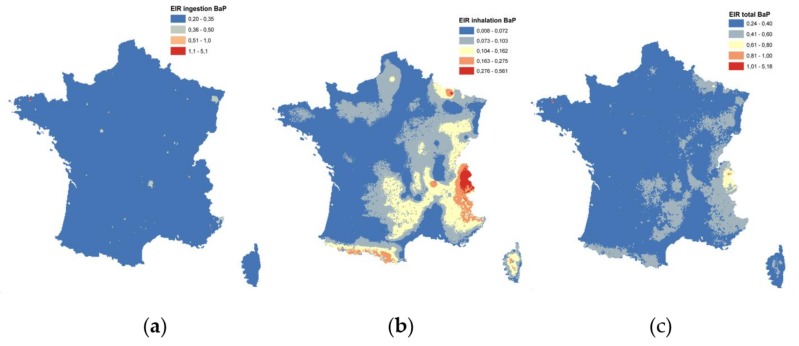
Excess of Individual Risk (×10^6^) for B[a]P for (**a**) ingestion (food, water, and soil); (**b**) inhalation, and (**c**) total in France.

The LADD variability between the modeled values, was a result of the contaminant concentration variation, combined with the different diet scenarios (self-consumption factor, type of food, and quantity ingested). The predominant media of exposure to Benzo[a]pyrene, for both classes, was the ingestion of commercial food, that did not include vegetables. The concentration of B[a]P in these products was considered the same, in the area of study, and stable, for the period examined. The second greatest contribution was from drinking water, followed by the consumption of vegetables, as a close second. Finally, the ingestion of soil had the smallest impact in the LADD. In this analysis, the EIR was used to aggregate ingestion and inhalation risk and compare the contributions (Figure 11). Overall, the EIR due to ingestion was significantly higher than that of the inhalation.

## 4. Discussion

The assessment of health risk associated with exposure to environmental hazards and the analysis of the spatial variation of risks, require, both, accurate and detailed population and environmental data, as well as clearly defined exposure assessments. Efforts were made to select complete, accurate, and up-to-date datasets, to include in the process of exposure assessment. In environmental modeling and the evaluation of environmental health inequalities, data from different sources were usually considered—emission sources, environmental quality, exposure, and population. Data were selected according to availability of harmonized databases and their geographical and temporal representativeness. Depending on the case, data that did not exactly meet the objectives of the study, could be adapted with appropriate processing, and constituted a robust base to assess the human health risk. 

To overcome the limitations imposed by the quality of the available databases, statistical and geostatistical processing techniques were employed, addressing the issues that arose from each environmental compartment, separately. Due to its hydrophobic nature, B[a]P was found in water in small concentrations, therefore, the exact measurement could not always be reported. Reconstructing these concentrations was not trivial, especially when the rate of observations under the detection limit was high. An imputation method was selected to treat those values under the detection limit to avoid distortion when estimating the contribution of the drinking water as an exposure pathway towards the total exposure. The imputation that was performed, considering the relationship between the B[a]P and other PAH substances, as well as the temporal correlation, allowed for capturing a more realistic spatial distribution, compared to a simple substitution method that would result in a smooth, uniform result. As a result, a refined spatial distribution of the B[a]P might also have reflected the pollution origins, and the variance between the imputed datasets provided a quantification of the associated uncertainty.

Environmental data were usually collected from monitoring locations, and therefore, were samples and did not provide sufficient spatial representativeness or exhaustive characterization of the environmental media. In many cases, the use of geostatistical methods that could consider several auxiliary/secondary variables were employed to address this issue. Combining complementary databases—such as the emissions, population, and altitude, in the case of air and the physio-chemical properties and distance from the polluted sites, for the case of soil—allowed for capturing the phenomena, more exhaustively. The relevance of the auxiliary variable(s), in each case, was evaluated before (correlation) and after (cross-validation) the modeling. Specifically, in the air compartment two different auxiliary variables were employed in the spatialization process of the measurements, for the two years. For 2010, altitude was included in the model, increasing the accuracy. The population was inserted in the model to limit the effect of the altitude for the mountainous areas, which were sparsely inhabited. For 2011, no strong correlation with altitude was observed. This difference between the two years could be explained by the differences in climatological conditions. For example, a particularly cold winter could increase the need for domestic combustions, in the areas of high altitude, and consequently lead to increased emissions of PAH. Since the available data corresponded to annual mean observations, it was not possible to account for this seasonal effect. For a more refined temporal approach, a seasonal modeling, taking into account the climatological data, could be established. Uncertainties and model limits were difficult to estimate in specific situations where no data were available (high altitude and low population density for air). To avoid the improper characterization of exposure, we defined area conditions of not-relevant estimations—altitude should be above of 1800 m and population density below 30/km^2^. Nonetheless, by combing the results of the two consecutive years to include in the exposure model, we minimized the effect of these extreme events that might appear in a year but not in the following. Depending on the data, it would be interesting to include other years of measurements, when such data are available, and study the sensitivity of exposure by inhalation to the considered time period.

Indicator kriging used for soil data processing, requires a strong assumption of stationarity that was not necessarily fulfilled by the data. Nevertheless, the use of this stationary model allowed us to gain some insight from data that would otherwise be disregarded. The use of a random-forest regression has previously shown to be a more successful prediction method than multiple linear regression, in the mapping accuracy [36]. Moreover, physio-chemical soil properties have also been shown to influence the spatial distributions of the B[a]P [42]. The random-forest regression selected to fit the residuals before the spatial interpolation allowed to include the various variables in the model (soil properties, distance from polluted sites, indicator kriging result), in a more flexible framework than linear regression, which was usually used. After calibrating the model, the random-forest residual kriging was shown to outperform linear regression. Random forest regression was also known to be more expensive, cost-wise, however given the size of the database and the improvement of the result, the difference was negligible. 

Starting with the available sparse spatial monitoring databases and with the appropriate processing, we assembled a refined spatial database to serve as an input in the spatial exposure model. Benzo[a]pyrene, which was the focus of this study, was a good indicator of the rest PAH substances, due to the strong correlation between them and the fact that it was found in higher concentrations than the rest. In this approach, an upstream environmental compartment was used as an input for the model, to predict the other, or was used as a secondary information to increase the data resolution. In the other direction of the chain, this allowed for estimating the contributions of the upstream compartments.

The results obtained, allowed us to identify the areas in which high exposures were more likely to occur. The similarity between the map of the exposure by ingestion (Figure 11a) and the spatialized concentration of the B[a]P in water (Figure 3) showed that the exposure due to ingestion corresponded mainly to the drinking water intake. However, by examining the map of the total EIR, the ingestion route was found to be the dominant contribution, but the spatial structure of the total spatialized risk indicator followed the same pattern as the spatialized atmospheric concentration of B[a]P in France.

Some hotspots correspond to high air concentration values, localized in the mountain areas, where air dispersion was limited. Concerning the ingestion, commercial products had the highest contribution towards the total exposure dose, with drinking water and vegetables following with similar contributions. Soil appeared to have the smallest contribution for the different age group scenarios. The highest hotpot due to water ingestion was localized in the proximity of an old gas factory.

It should be noted that, by ingestion, we refer to the intake through soil, water, and aliments consumption, and it included the possible contamination of B[a]P due to the cooking process. Regarding inhalation, smoking, as evidence suggests, is a determinant factor of exposure [43], however, it was not considered in this study, due to the lack of data. Dermal exposure can also occur by direct skin contact with consumer products of a category of petroleum, such as lubricants and coatings in rubber boots, was also not considered. A complete study that considers all the remaining exposure routes and extends in the calculation of the internal dose as well, could be found in the work of D. Sarigiannis et al. [44] where dermal pathway was insignificant, in comparison with the ingestion or inhalation pathways.

This refined approach to exposure characterization allowed us to identify significant differences experienced by the different age groups, as well as people living in different areas in France. Moreover, these aggregated exposure maps could also be of help to concentrate sampling campaigns, in areas with poor information density or features of great interest. The use of GIS allowed us to map the environmental health inequalities of exposure, on a fine resolution, by spatializing the risk indicators. The spatial structure of risk maps reflected the influence of a complex set of spatial and environmental factors, with great variations, and operated in different spatial scales (Figure 11). The analysis of these structures allowed us to quantify the scope and the contribution of the different scales of spatial variability.

**Figure 12:**
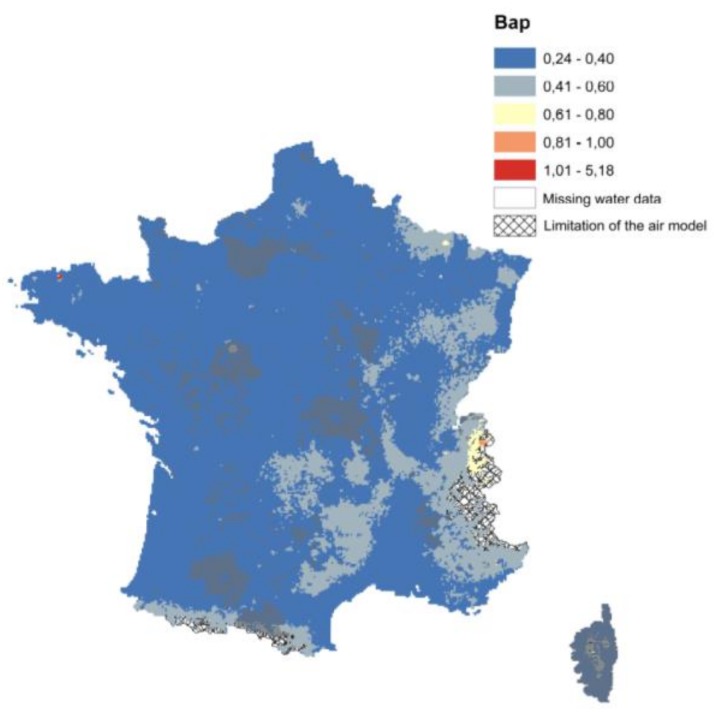
Final map of the B[a]P Excess of Individual Risk (EIR) (×10^6^) including the model’s limitations.

A way to report the uncertainties on a map could be in the form of intervals. In this regard, the use of ranges of values for a particular metric of decision-making relevance (e.g., range of uncertainty associated with a particular estimate of exposure), might be adequate, as long as the bounds of the ranges are properly placed with a probabilistic context (e.g., 95% confidence intervals, interquartile range). One other approach that we tested was to present a map with an adapted grid size related to data precision or population density. Finally, we have chosen to add additional information on the total EIR map (Figure 12).

- Grey borders were associated with grids where water data were missing (no data).
- Black cross-hatch was used to hide areas outside of the validity limit of the air statistic model.

This information should be clearly reflected in the presented results, when the associated uncertainty was too high for the results to be trustworthy. In this way, decision makers are ought to understand whether their decisions are likely to be improved by waiting for additional information on critical factors influencing these decisions, or regarding the need to consider an adaptive strategy, based on iterative reassessment.

## 5. Conclusions

Spatial identification of environmental health inequalities, their determinants, and susceptible populations were studied through the integration and processing of spatial data to construct exposure indicators:

- GIS provided geoprocessing tools to assembled databases from different sources;
- developed statistic methods improved the spatial and temporal data representativeness;
- the exposure multimedia linked the environmental and exposure compartments;
- the exposure assessment framework provided a methodological framework to combine and integrate all exposure pathways.

The data processing and spatial predictions, unavoidably, introduced uncertainty in the model. Quantifying this uncertainty was important and should have accompanied the results, to avoid potential misclassification of areas and populations at risk of overexposure. 

An exhaustive description of exposure required the integration of data from different environmental compartments and supports. However, limitations due to data availability or quality usually arose. The use of these databases was not initially assembled for the objective of estimating exposure, which might have led to biases in the estimations. Common problems included limited number of observations, spatial and temporal heterogeneity, lack of meta-data, and measurements under the detection limit. In this work, to address the issues that arise from the poor quality available data of the B[a]P, in the three environmental compartments, statistical and geoprocessing techniques were employed. Specifically, temporal correlation and relations between the variables were employed in an effort to make up for the under the detection limit values. Moreover, complementary databases were used to construct interesting auxiliary variables to improve the spatial data representativeness. The refined spatial databases assembled then provided a more robust base to assess the human exposure. Our results led us to identify the pollutant sources, determinants of exposure, and potential hotspot areas. In this way, areas and population in risk of overexposure could be identified early, allowing the public authorities to implement policies to manage the phenomena. Exposure indicators and data processing algorithms will be integrated in the French coordinated integrated environment and the health platform PLAINE, to map and analyze the environmental health inequalities, at the national scale, for other substances (such as pesticides).

## Figures and Tables

**Table 1:** Available data and support.

Variable	Original Data Resolution and Support	Source and Use
Atmospheric concentration	Measurement points	National air quality database (2010 and 2011 data). To be interpolated using regression kriging.
Atmospheric emission	Grid 0.01° × 0.01°	Annual emissions of the National Spatialized Inventory (2007 and 2012). To be processed as auxiliary variable for the calculation of atmospheric concentrations.
Altitude	Centroids of French municipalities	National inventory. Aggregation from raster using the area ratio. To be processed as auxiliary variable for the calculation of atmospheric concentrations.
Drinking water concentration	Surface (municipalities)	Multi-annual measurements of SISE’Eaux database (years 2000–2012). Multiple imputation method to include values under the detection limit and spatialization according to the drinking water network.
Population	Grid 1 × 1 km	Aggregation by surface ratio to be used as an auxiliary variable for the calculation of atmospheric concentrations.
Topsoil concentration	Grid 16 × 16 km	RMQS [23] (French soil monitoring network) for year 2010. To be interpolated using residual kriging.
Polluted sites	788 points	BASOL (National database of polluted or potential polluted soils). To be used as an auxiliary variable in spatializing topsoil concentrations.
14 Soil properties	Grid 3 × 3 km	INRA. To be used as auxiliary variables in spatializing topsoil concentrations.

**Table 2:** Absolute Mean Error (AME) and Root-Squared Mean Error (RMSE) obtained for the imputation and the naive methods.

DL Threshold	Method	AME	RMSE
	Imputation	0.17	0.37
3 µg·L^−1^	DL/2	1.18	1.29
	DL/√2	1.37	1.49
	Imputation	0.21	0.46
5 µg·L^−1^	DL/2	1.95	2.12
	DL/√2	1.74	1.89
